# Synthesis of a graphene oxide/agarose/hydroxyapatite biomaterial with the evaluation of antibacterial activity and initial cell attachment

**DOI:** 10.1038/s41598-022-06020-1

**Published:** 2022-02-04

**Authors:** Ingrid Patricia Khosalim, Yu Yuan Zhang, Cynthia Kar Yung Yiu, Hai Ming Wong

**Affiliations:** Paediatric Dentistry and Orthodontics, Faculty of Dentistry, The University of Hong Kong, Hong Kong, SAR China

**Keywords:** Health care, Medical research

## Abstract

Various materials are used in bone tissue engineering (BTE). Graphene oxide (GO) is a good candidate for BTE due to its antibacterial activity and biocompatibility. In this study, an innovative biomaterial consists of GO, agarose and hydroxyapatite (HA) was synthesized using electrophoresis system. The characterization of the synthesized biomaterial showed that needle-like crystals with high purity were formed after 10 mA/10 h of electrophoresis treatment. Furthermore, the calcium-phosphate ratio was similar to thermodynamically stable HA. In the synthesized biomaterial with addition of 1.0 wt% of GO, the colony forming units test showed significantly less *Staphylococcus aureus*. Initial attachment of MC3T3-E1 cells on the synthesized biomaterial was observed which showed the safety of the synthesized biomaterial for cell viability. This study showed that the synthesized biomaterial is a promising material that can be used in BTE.

## Introduction

The increasing interests in research on regenerative medicine and tissue engineering have prompted the development of their use in clinical practice^1^. Bone Tissue Engineering (BTE) is one field that has been developing rapidly for several decades. BTE mainly focuses on enhancing bone regeneration and repair by creating substitutes to traditional bone grafting materials^2^. However, clinical applications of engineered constructs are often limited due to poor biocompatibility or mechanical properties of the developed materials^3^.

Clinical application of BTE in dentistry includes socket preservation^4^, alveolar ridge augmentation^5^, and guided bone regeneration^6^. Natural materials, synthetic materials, bioceramic and metals have been used in BTE. However, these materials are also associated with considerable drawbacks^7^. Natural materials such as collagen, chitosan, and alginate have weak mechanical properties, fast degradation time and lack of bioactivity required for hard tissue formation^8^. Synthetic materials such as polycaprolactone, polylactic acid, polyglycolic acid, and poly lactic co-glycolic acid release acidic products during degradation and may cause necrosis of tissue^9^. Bioceramics such as calcium phosphate bioceramic, hydroxyapatite (HA), β-tricalcium phosphate, and bioactive glass are difficult to shape due to extreme brittleness, stiffness, low flexibility and molding property, and weak mechanical^10^. Metals such as titanium alloy, magnesium and its alloy do not have the potential for drug delivery, do not degrade (except magnesium and its alloys), and lack of bioactivity^11^.

The natural bone is composed of non-mineralized organic component (predominantly type-1 collagen) and mineralized inorganic component that is, carbonated apatite minerals^9,12^. In BTE, the ideal scaffold should be analogous to the natural bone and provide a favorable microenvironment for cell growth^13^. To imitate the composition of natural bone, macromolecules as the organic substrates and HA as the inorganic phase are mixed in appropriate proportions to mimic the organic − inorganic composition of bone tissue^14^.

Graphene materials have been used in biomedical application because of good biocompatibility, strong mechanical strength, high elasticity and good flexibility^15^. Graphene oxide (GO) is an emerging luminescent carbon nanomaterial and has been widely explored^16^. GO is an oxidized derivative of graphene and has many hydrophilic functional groups, such as hydroxyl and carboxyl groups, that present high dispersibility in aqueous solutions and can serve as anchor sites for binding with metal ions or nanoparticles^17^. Previous studies have shown that GO can enhance both the mechanical properties of the substrates and the cellular behavior^18,19^. GO substrates also can also promote proliferation and differentiation of various cell lines, including induced pluripotent stem cells^20^, C2C12 myoblasts^21^, PC12 cells^22^, and mouse^23^ as well as human^24^ mesenchymal stem cells. Therefore, GO can act as an effective reinforcement in scaffold materials to improve their biological properties^25,26^. A study by Mazaheri et al., observed that low concentration of GO resulted in biocompatibility and kept the mechanical flexibility of the self-sterilized layers for high proliferation of human mesenchymal stem cells^27^. The other marked advantage of GO is its antibacterial activity, which is an important characteristic of scaffold in BTE to prevent or control infection^28^. Many studies have proved its antibacterial activity against *E. coli*^29^, *P. aeruginosa*^30^, *Streptococcus mutans, Porphyromonas gingivalis, Fusobacterium nucleatum*^31^, *P. syringae, X. campestris pv., F. graminearum, F. oxysporum*^32^, *Bacillus subtilis, Enterococcus faecalis,* and *Salmonella typhimurium*^33^.

Agarose (AG) is a natural polymer widely used in BTE. It is biocompatible and biodegradable natural polysaccharide (made of repeating unit of agarobiose) which exhibits similarity to bone extracellular matrix. Agarose has the ability to form a gel network allowing diffusion and transport of oxygen and nutrients within the scaffold^34^. The stiffness of AG hydrogels is controllable, which allows the manipulation of the mechanical properties of the scaffold^35^. Agarose is suitable as an alternative organic matrix; however, it is known to be unfavorable to cell adhesion, thus it is often combined with other polymers to improve its biocompatibility^34,36^.

Hydroxyapatite is a bioactive inorganic ceramic [Ca_10_(PO_4_)_6_(OH)_2_] with chemical and crystallographic similarity to the natural apatite in bones^37^. It is the main mineral component in bone and stable form of calcium phosphate. HA is an effective component for biomaterials, because of its good biocompatibility, osteogenic activity, biodegradability and good cell adhesiveness^38^. Although HA is a conventional material for bone scaffold, it is brittle, and difficult to shape. Thus, HA is often combined with other materials such as metal, polymer and others to form composite^39,40^.

We developed a biomaterial with key components of GO, AG and HA in this study. To date, there has been no report of bone tissue scaffold material fabricated with triple component of GO/AG/HA in the literature. In BTE, most researchers produced highly porous biomaterials by either application of gas foaming agent and freeze-drying method separately or by the use of advanced and expensive techniques, such as 3D printing^41^, and electrospinning^42^. In this study, we also introduced simple and cost-effective electrophoresis system to produce biomaterials. Electrophoresis enables ion migration in a specific one-dimensional direction. It can transport ions more rapidly through a gel or solution than diffusion alone and can be used to accelerate HA formation in agarose hydrogels for the synthesis of HA-agarose hybrid materials^43–45^. The aim of this study was to investigate whether the synthesized biomaterial containing GO/AG/HA can be used as a suitable candidate for BTE.

## Results

### Characterization of synthesized biomaterials

**Figure 1:**
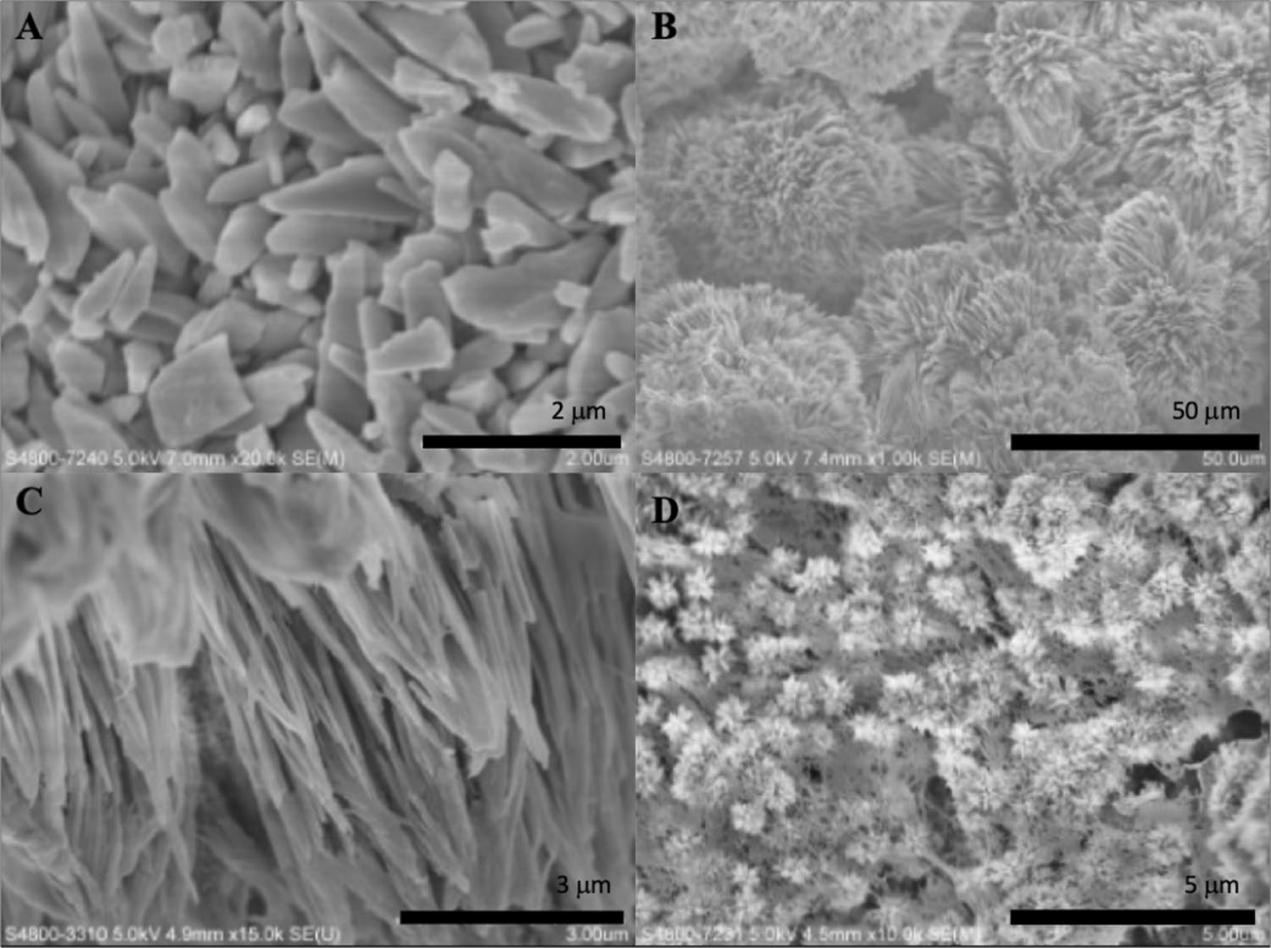
SEM micrographs of formed crystals treated with different strength of electric current. (**A**) 5 mA; (**B**) 8 mA; (**C**) 10 mA; (**D**) 12 mA.

**Figure 2:**
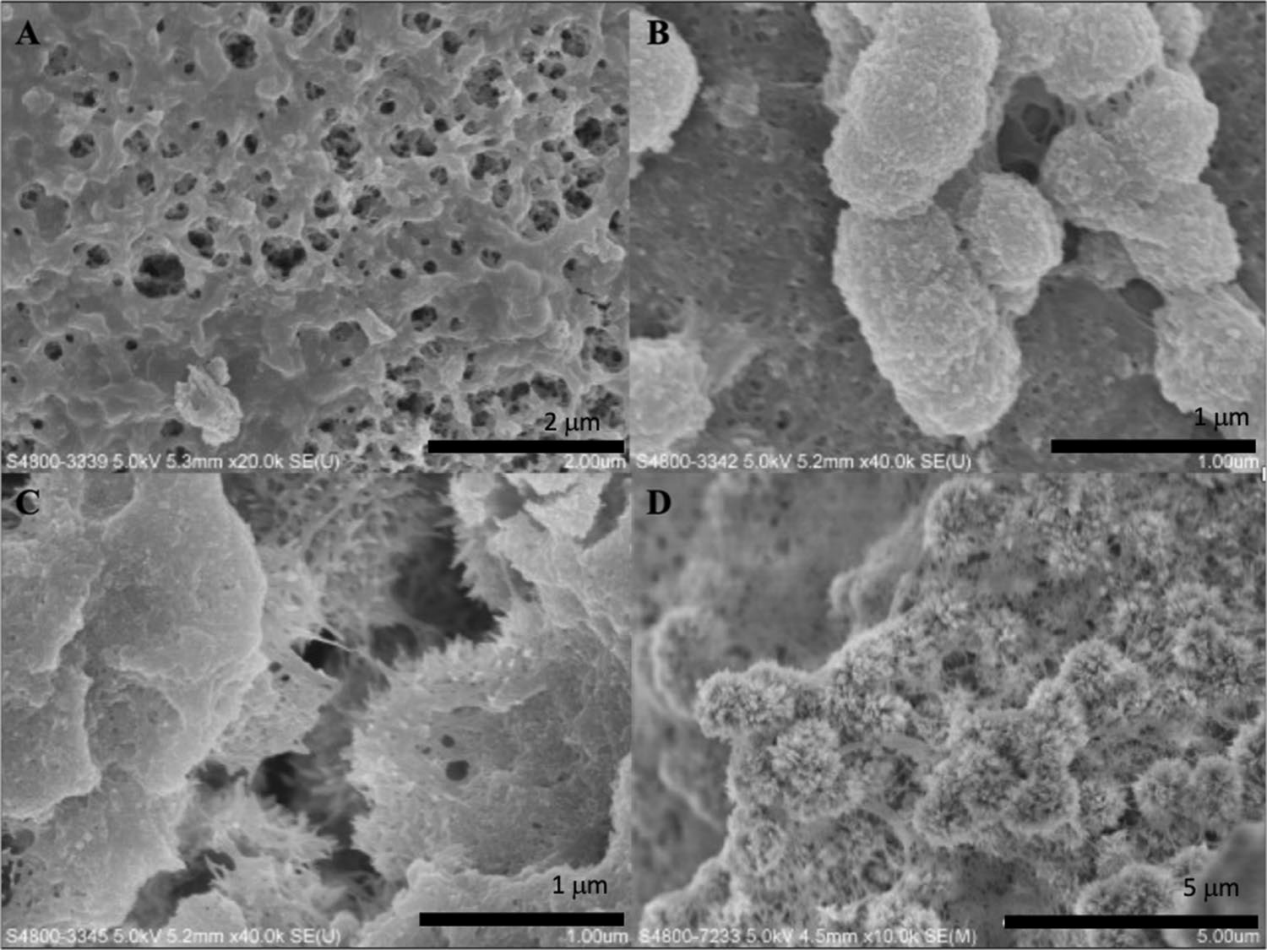
SEM micrographs of synthesized biomaterials treated with a current of 10 mA, after different mineralization time. (**A**) 3 h; (**B**) 4 h; (**C**) 6 h; (**D**) 8 h.

**Figure 3:**
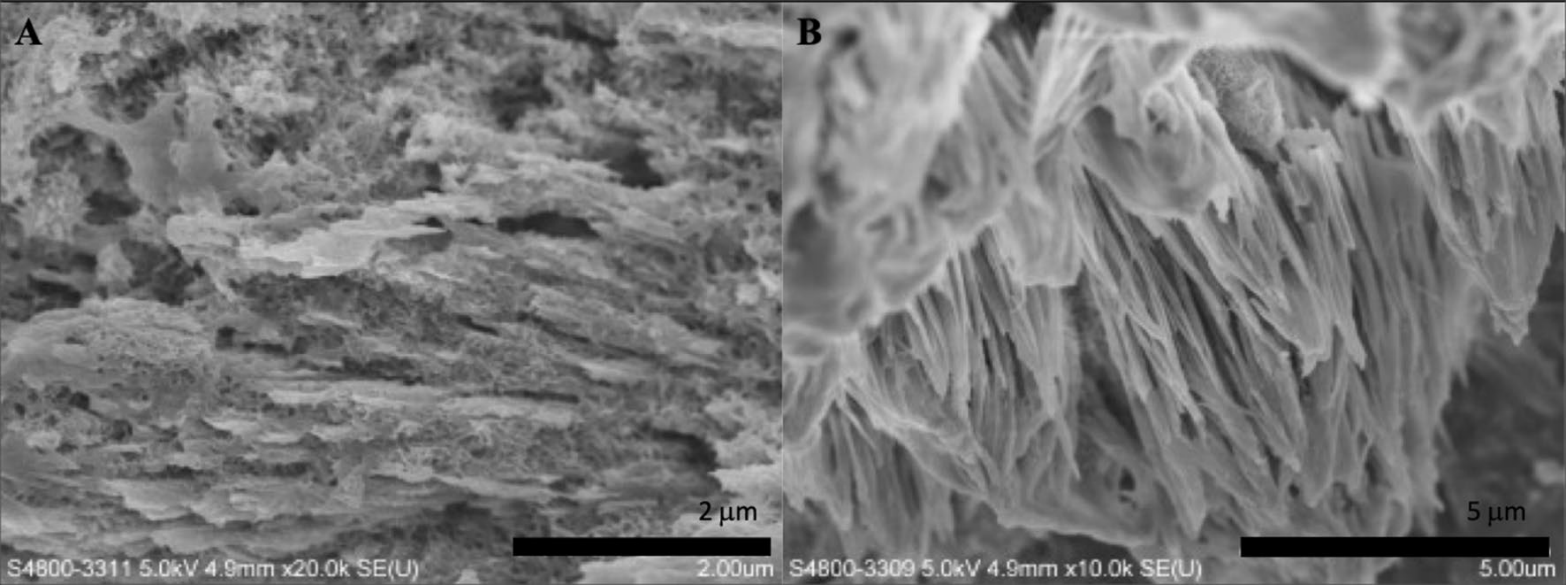
SEM micrographs of the synthesized biomaterial treated with a current of 10 mA, after 10 h mineralization. (**A** 20.0 k × magnification; **B** 10.0 k × magnification).

Scanning electron microscopy was used to examine the surface of the synthesized biomaterials. In this study, we found that crystals with different morphology were formed when different strength of electric current was applied. When the applied electric current was 5 mA, 8 mA, 10 mA and 12 mA, polygon-shaped crystals (Fig. 1A), flake-like crystals (Fig. 1B), needle-like crystals (Fig. 1C) and flower-like crystals (Fig. 1D) were formed, respectively. Figure 2 showed the surface of the synthesized biomaterials treated with different duration of mineralization. When the synthesized biomaterial was mineralized for less than 5 h, organic substance was the primary component formed. The SEM showed that formed crystals were wrapped with a layer of gelatine. When the mineralization time increased, more crystals were formed and the synthesized biomaterials became harder. After 8 h of mineralization, needle-like crystals were observed on the synthesized biomaterials (Fig. 2D). With increasing mineralization time, more crystals were formed. The crystal growth might be inhibited when HA crystals came into contact with one another, and finally, the crystals grew parallel to one another (Fig. 3).

**Figure 4:**
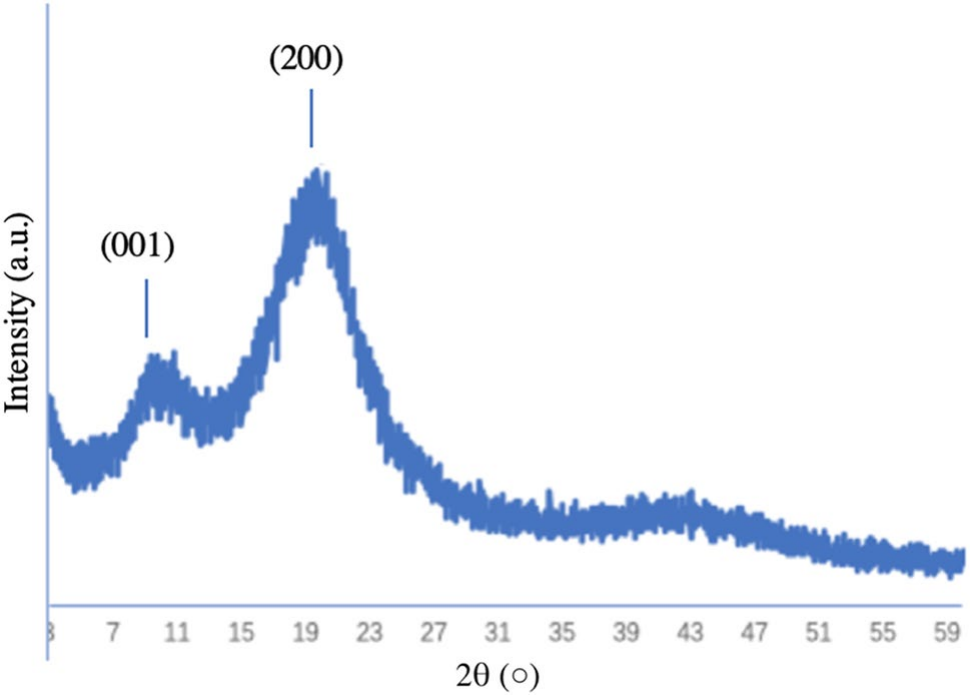
XRD spectrum of the synthesized biomaterial treated with a current of 10 mA, after 10 h mineralization.

X-Ray Diffraction is a method based on the constructive interference of monochromatic X-rays and crystal samples. In this study, the XRD analysis was performed on the samples treated with 10 mA/10 h electrophoresis with addition of GO (Fig. 4). The XRD spectrum showed orientation of formed crystal bunches with the crystal plane (001) at 2θ = 9.3° of GO and (200) at 2θ = 22.1° of hydroxyapatite.

**Figure 5:**
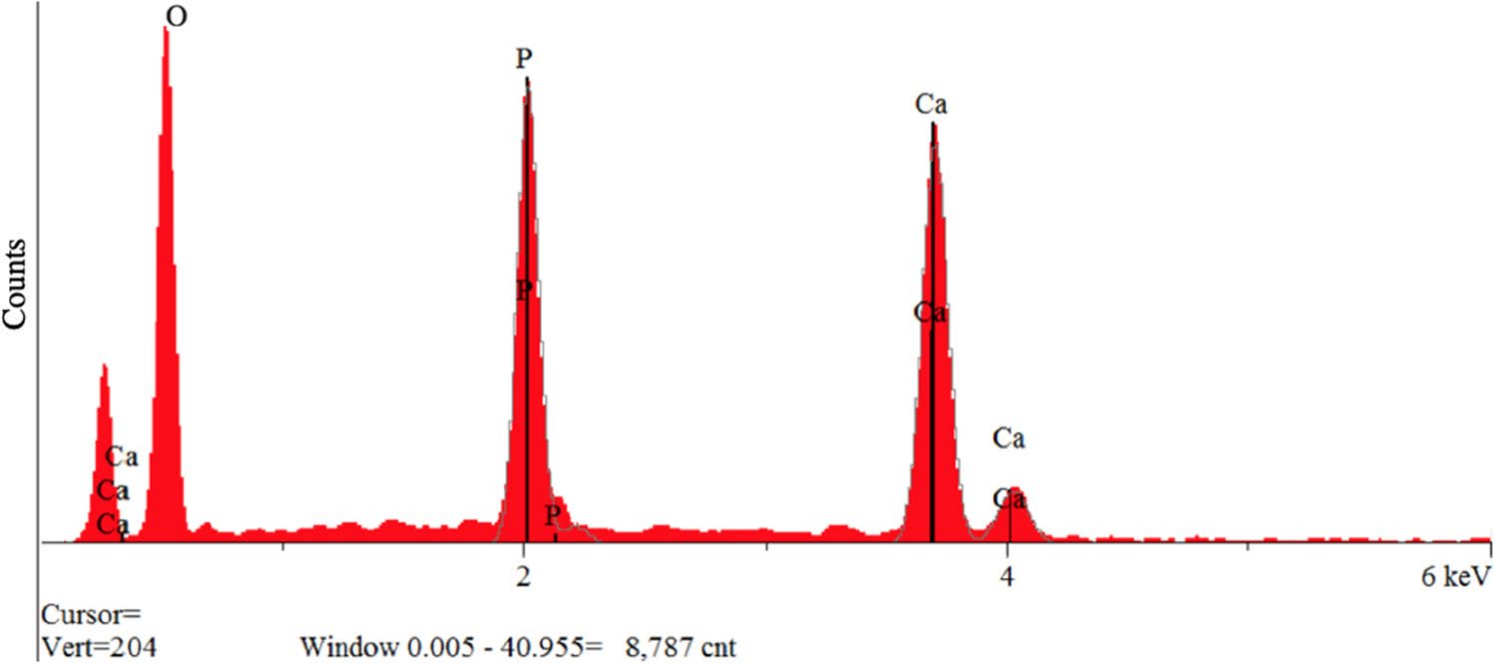
EDS spectrum of the synthesized biomaterial treated with a current of 10 mA, after 10 h mineralization.

Energy dispersive spectroscopy (EDS) was applied to measure the Ca/P ratio of the synthesized materials (Fig. 5). The EDS data showed that after 8 h of mineralization, the mean Ca/P ratio of synthesized materials was 1.68 ± 0.045.

### Evaluation of antibacterial activity

**Figure 6:**
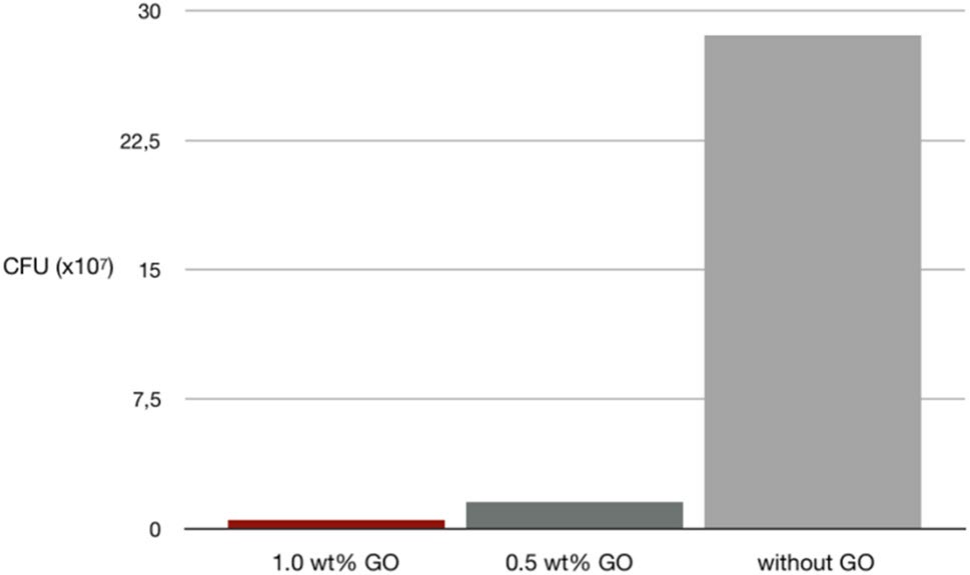
CFU counting evaluation of synthesized biomaterials with addition of 1.0 wt% GO, with the addition of 0.5 wt% GO, and without the addition of GO.

The antibacterial properties of the synthesized biomaterial on *Staphylococcus aureus (S. aureus)* strains were evaluated by colony forming units (CFU) method. After 24 h of incubation, the quantity of adherent bacteria on the synthesized biomaterials with the addition of 1 wt% GO ((0.52 ± 0.17)*10^7^ CFU/ml) were significantly less than that on the synthesized biomaterials with the addition of 0.5 wt% GO ((1.55 ± 0.21)*10^7^ CFU/ml; *p* = 0.0026, t-test) and the synthesized biomaterials without the addition of GO ((28.60 ± 2.20)*10^7^ CFU/ml; *p* < 0.0001, t-test) (Fig. 6). The quantity of adherent bacteria on the synthesized materials with the addition of 0.5 wt% GO were also significantly less than that on the synthesized materials without the addition of GO (*p* < 0.0001, t-test).

### Evaluation of initial attachment

**Figure 7:**
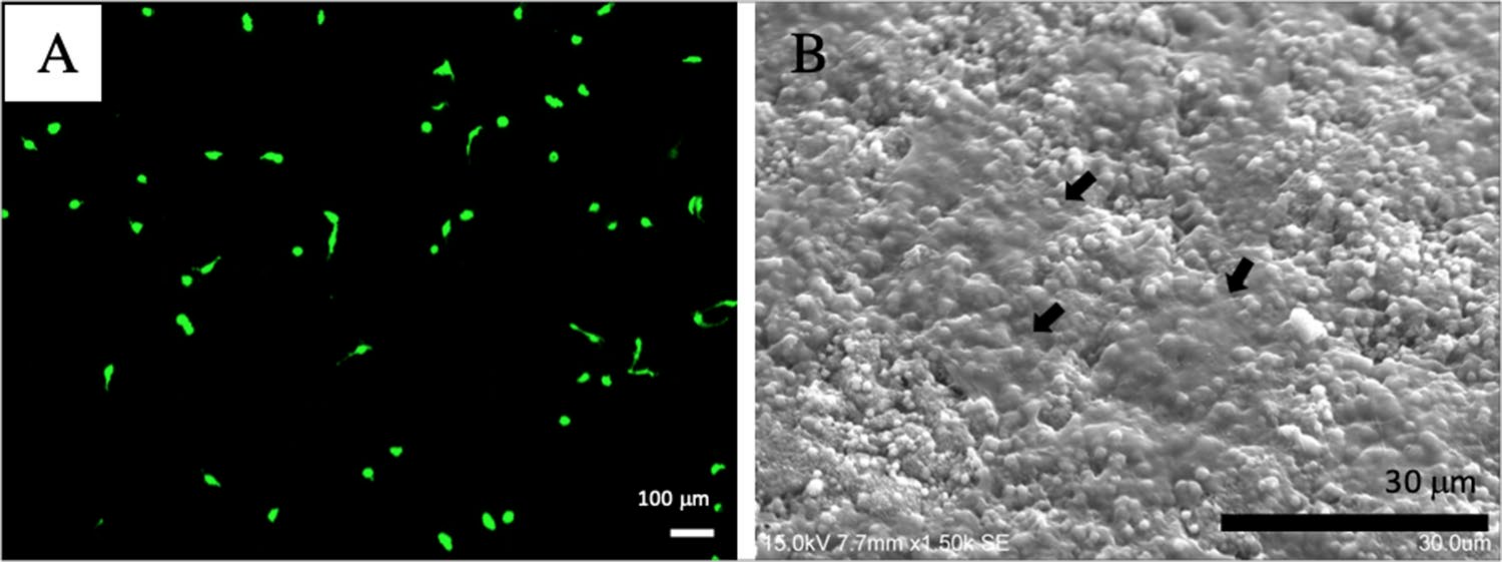
MC3T3-E1 cultured on synthesized biomaterial after 3 days on (**A**) LCSM; (**B**) SEM, with the arrows represent attached MC3T3-E1 cells.

Cellular morphology was evaluated using laser scanning confocal microscope (LSCM). The morphology of MC3T3-E1 was assessed after it was cultured on the synthetized biomaterial after 3 days (Fig. 7A). It was observed that the cell morphology on the synthesized biomaterial was preserved, showing shutter-like and polygon-shaped cells.

SEM images of the cells after 3 days of culture on the synthetized biomaterial was also obtained to observe the interaction. The SEM micrograph reflected the status of MC3T3-E1 cells attachment after 3 days of culture (Fig. 7B). It was observed that the MC3T3-E1 cells were attached, and the surface of the synthetized biomaterial was mostly covered by the cells.

## Discussion

Crystal growth could be controlled and regulated by organic template, character of the substance surface and addition of surfactants. In the initial stage of crystallization process, the crystal nucleation, morphology and orientation were dominated by gelatine because of the constant electric current. The synthesized biomaterial was hybrid which mainly composed of organics. Thus, formed crystals were covered by a layer of gelatine, and their morphology was hardly detected (Fig. 2B and C).

In Fig. 3, it was observed that HA crystals grew parallel to one another. The process of initial HA crystal growth required time. The HA crystal adapted and self-adjusted, gradually formed a parallel array along the c-axis. After completing the self-adjusting process and more crystals formed, the regulation of gelatine on crystal growth decreased, especially the crystals located away from the gelatine matrix.

X-ray diffraction has become a common technique in the research field of crystal structure and atomic spacing. The X-rays were generated by the cathode ray tube, filtered to produce monochromatic radiation, collimated and then directed to the sample^46^. XRD analysis is an excellent method that has been widely used to study the ultrastructure of certain materials. XRD is also a powerful technique that can be used to evaluate the structure of biological minerals such as teeth and bones, and has been used by various researchers to examine the crystal structure of biological mineral composites^47^. In this study, the XRD result showed no impurity peaks, indicating that the synthesized biomaterial had high purity. In the earlier stage of mineralization process, the crystals grew in all direction from the nucleation sites, as shown in Fig. 2C and D. Due to limited space available between adjacent sites of growth, only perpendicularly growing crystals could effectively utilize the available space, resulting in the formation of parallelly oriented crystals^48^. Therefore, it was observed that after the formation of un-oriented HA crystals, crystals growth take place in the available free space, producing crystals growing perpendicular to the surface of un-oriented HA crystals (Fig. 2D).

The result of the EDS showed that the synthesized material had a similar calcium/phosphate ratio with that of thermodynamically stable HA (Ca/P = 1.67). In the EDS method, analytical information is derived from depths that are generally typical for thin film analysis. The particle size affects both qualitatively and quantitatively the ratio of Ca and P. As the accelerating voltage increases, the signal strength increases corresponding to the Ca and P elements (up to 15 kV) regardless of the particle size of the Ca/P. In the 15–30 kV range, shape variation is observed for particle sizes with minimum and maximum dimensions, and is uniform in the median case^49^.

In a previous study, it was investigated that OH^−^ could regulate crystal growth by triggering the active sites at crystal facets^50^. Specifically, the pH determined whether crystal growth was affected by the crystal’s interior structure or by the exterior mineralization condition. The exterior mineralization condition dominated the crystallization when the pH value was higher. In the electrophoresis-aided mineralization system, the electrochemical reaction caused the water in the vicinity of the cathode hydrolysed. This process resulted in increasing pH due to hydroxyl aggregation. The increase of electric current strength could accelerate the electrochemical reaction, leading to more hydroxyl formation and the increase of local pH value. Thus, different crystal morphologies were observed when different electric current strengths were applied.

Besides, at a higher OH^−^ concentration, each crystal facet generates more active sites^51^. With the increased concentration of OH^−^ ions, ion aggregation became faster and Ca/P clusters with ‘tentacles’ formed^52^. This process resulted in crystals with small branches at their ends. The single needle-like crystal was formed when the applied electric current was 8 mA; while, crystals with branches were formed when the applied electric current was 10 mA.

*S. aureus* is a Gram-positive, non-motile, non-spore forming grape-like cluster. It is the most important coagulase-positive pathogen of staphylococci due to its combination of toxic-mediated virulence, invasion and antibiotic resistance. *S. aureus* is a common pathogen found in various oral diseases, such as oral mucositis, periodontitis, peri-implantitis, endodontic infections and even dental caries. Despite its pathogenic potential, *S. aureus* is rarely associated with acute dento-alveolar infection. Other oral infections that have been associated with *S. aureus* include infected jaw cysts, oral mucosal lesions and denture-induced stomatitis^53,54^.

Effect of graphene-based materials on microbial cell structure, metabolism and viability have been investigated. The effectiveness depends on the concentration of the substance, exposure time, physico-chemical properties, as well as the characteristics of the microorganisms used in the test. There are various ways for graphene-based materials to destroy the microbial cells, including disruption of cell walls and membranes through sharp edges of GO; generation of reactive oxygen species, which can be a degradation factor for microbial cells. In the study of Thani et al., it showed that GO has antibacterial and antifungal activity against microorganisms. The anti-microbial activity of GO was detected by a spectrophotometer as an indirect method to measure the growth and cell count against these microorganisms, one eukaryotic fungus (*Candida albicans*) two Gram negative bacteria (*Escherichia coli* ATCC 41570 and *Pseudomonas aeruginosa* ATCC 25619) and two Gram positive bacteria (*Streptococcus faecalis* ATCC 19433 and *S. aureus* ATCC 11632)^55^.

The unique properties of GO and reduced-GO can be used clinically for broad-spectrum antimicrobial disinfection treatment. There are a few known mechanisms related to the antibacterial activity of GO-based materials, such as: interaction of extremely sharp edges of GO sheets with wall membrane of the bacteria^56^, charge transferring^57^, wrapping the bacteria within the aggregated GO sheets^58^, reactive oxygen species generation^59^, inhibiting the bacterial respiration^60^ and/or the glycolysis processes^61^, RNS generation^62^ and DNA fragmentation^63^. Antibacterial activity of GO-based materials depends on the material’s properties, such as size, solubility, dispersion, duration of interaction and concentration, density of functional groups and the production of cellular oxidative stress^64^. Graphene materials of smaller size (e.g. GO) have higher cytotoxicity compared to those with larger size. Antibacterial activity of GO has been attributed to membrane stress induced by sharp edges of graphene nanosheets. GO nanosheets can go through the cell membranes of bacteria and intensely extract great amounts of phospholipids from the membranes. GO nanosheets also can oxidize glutathione, which acts as redox state mediator in bacteria^31,59^.

The study of Romero et al. showed positive result of using GO on Gram-negative *Escherichia coli* and Gram-positive *S. aureus.* The study concluded that GO and reduced-GO can be used in dermatological infections, because the effect on human skin fibroblasts from these treatments is low compared to the antibacterial effect^65^. Study by He et al., showed that GO was effective in killing dental pathogens, both Gram-positive and Gram-negative bacteria^31^. In this study, after the introduction of GO, the synthesized biomaterial showed the antibacterial property to *S. aureus*. In the group with 1.0 wt% GO, the CFU count of the *S. aureus* was found significantly less than 0.5 wt% GO.

Cell morphology greatly affects various cellular events, such as proliferation, differentiation, cytoskeletal organization, and gene expression. MC3T3-E1 cells are well-known in vitro osteogenic model system and have been widely used in BTE-related research. MC3T3-E1 cells display a sequential developmental pattern of proliferation and differentiation, resulting in calcified bone tissue similar to bone formation^66^.

The study by Yang et al. showed that dose-dependent upregulation of protein-positive green fluorescent green dopamine neurons increased by threefold when treated with 100 g/ml GO, while exposure to 1 g/ml GO did not show significant promotion compared without nanoparticle control and graphene or carbon nanotubes^67^. Another study by Alegria et al. demonstrated an enriched hemangioblast cell population when cultured in GO cover slips compared to standard gelatin-coated plates as control. The cell morphology observation on day 1 revealed increased cell cluster formation in GO-cultured group compared to controls^68^. Another study by Mazaheri et al., used GO-chitosan composite layers to evaluate antibacterial activity and cell proliferation. Significant antibacterial activity against *S. aureus* was observed and the surface density of the human mesenchymal stem cell cultured on 1.5 wt% GO-chitosan was nearly the same. Their study found that at higher concentration of GO (6 wt%) could decelerate the proliferation of human mesenchymal stem cells^27^. In the present study, the synthesized biomaterial contained 1.0 wt% GO and it was observed that the addition of GO at this concentration improved the biological properties of synthesized biomaterial, allowing the initial attachment of MC3T3-E1 cells.

Agarose is a biocompatible and biodegradable material. It enables the diffusion and transport of oxygen and nutrition, thus allowing cells growth. However, it is unfavorable to cell adhesion^34^. In the synthesized biomaterial, HA enabled the MC3T3-E1 cells to adhere on its surface. HA is a bioactive and biocompatible material. As a major inorganic component of hard tissues, it has been widely used as a scaffold for mineral-associated tissue engineering and as a carrier for several growth factors. HA may be able to maintain optimal osseointegration over time, despite the molecular mechanisms^69^.

## Methods

### Synthesis of GO

Two GO solutions (0.5 and 1 wt%) were prepared separately by first heating 2.5 and 5 g, respectively, of critic acid powder (Sigma-Aldrich, St. Louis, MO). The citric acid was heated to 200 °C, and the color changed from colorless to yellow after 5 min. The color then changed to orange after 30 min and the heating was kept until it turned into black liquid after 100 min. The transformation into black liquid suggested the formation of GO^70^. This liquid was then added to 500 ml deionised water. Finally, the pH was adjusted to 5.5 with 1 M NaOH solution.

### Preparation of metastable mineralization solution containing GO

The 0.5 and 1 wt% GO solutions were separately mixed with 500 ml metastable calcium phosphate solution (5.8 mM Ca^2+^, 3.5 mM PO_4_^3−^, 1.17 mM F^−^) to form the metastable mineralization solution, containing 0.5 and 1 wt% GO, respectively. The pH of the mineralization solution was adjusted to 5.5 with 0.1 M HCl and 0.1 M NaOH. The solution was stored at 4 ℃ before use.

### Preparation of medium in agarose hydrogel

A CaCl_2_-agarose hydrogel was prepared by mixing 1.0 g agarose powder (Regular Agarose G-10, BIOWEST, Nuaille, France) into 100 mL of a 0.13 M CaCL_2_ solution (CaCl_2_ · 2H_2_O, Sigma-Aldrich, St. Louis, MO, USA). A GO-agarose hydrogel was prepared by mixing 1.0 g agarose powder into 100 mL of phosphate-containing-GO solution. The pH value of the solutions was adjusted to 6.5 using 0.1 M NaOH and 0.1 M HCl. The mixtures were enhanced for 30 min and then heated to 100 °C until agarose was completely dissolved.

### Fabrication of biomaterial by electrophoresis

CaCl_2_ agarose hydrogel and phosphate-containing-GO agarose hydrogel were put into the two sides of the tube. The tube was then connected to the plastic cells. Electrodes were set into the bottom of the cells, which were filled with 0.9% NaCl solution to enhance the electrical conductivity. The electric current was maintained constant at 5, 8, 10, 12 mA during electrophoresis. The gels and NaCl solution were refreshed every 2 h, and their exchange defined the completion of a cycle. The electrophoresis process was terminated after 5 cycles. The layer formed in the middle was harvested and freeze-dried overnight.

### Characterization and evaluation of synthetized biomaterial

The surface morphology and the chemical analysis with respect to the calcium/phosphate (Ca/P) ratio of the synthetized biomaterial were evaluated using field-emission scanning electron microscopy (SEM) and energy dispersive spectroscopy (EDS) (Hitachi S4800, Hitachi Ltd., Tokyo, Japan), respectively. The structure of scaffold was identified using X-ray diffraction (XRD) (X'Pert Pro, Philips Almelo, Netherlands).

### Evaluation of antibacterial activity

The antibacterial activity of synthesized biomaterials on *S. aureus* strains was evaluated using Colony Forming Units (CFU) counting method. The synthesized biomaterials (containing 1.0 wt% GO: n = 10; containing 0.5 wt% GO: n = 10; and without the addition of GO: n = 10) with a size of 3*3*1.5 mm^3^ were prepared and autoclaved to sterilize. *S. aureus* (ATCC 6538) cells at a concentration of 10^6^ CFU/mL was prepared. Sample with addition of 300 μL of *S. aureus* (10^6^ CFU/mL) was put in 1 mL tube. After incubation at 37 °C for 24 h, samples were vigorously vortexed for two minutes. One hundred μL from each sample was collected and immediately put in 900 μL brain heart infusion to dilute the bacteria concentration in tenfold. The diluted bacterial suspension corresponding to each sample was transferred into a horse blood agar. All the plates were incubated at 37 °C for 24 h and the number of CFU on the plate was counted visually.

The data collected in this study was quantitative, numerical, and directly measured by counting visible *S. aureus* coliform colonies on plates. Data are presented as mean ± standard deviation. The values of experiment groups are compared to those of the control groups. Differences between two mean values were calculated by paired t-test with statistic software (SPSS Statistic 24; IBM). Differences were considered significant at p < 0.05.

### Evaluation of initial attachment

MC3T3-E1 cells (obtained from the Cell Culture Centre of the Institute of Basic Medical Sciences Chinese Academy of Medical Sciences, China) were cultured in high-glucose DMEM supplemented with 10% fetal bovine serum, 100 U mg mL^−1^ penicillin, and 100 mg mL^−1^ streptomycin. The culture conditions were maintained at 37 °C in a humidified atmosphere containing 5% CO_2_ and the medium was renewed every 2 days.

For cell morphology and attachment evaluation, the synthesized biomaterial treated with 10 mA /10 h of electrophoresis system and with addition of 1.0 wt% GO was prepared to match the inside diameter of a 24-well cell culture plate. The synthesized biomaterial was sterilized by immersing in 70% alcohol for 30 min, and seeded with MC3T3-E1 cells at 3.5 × 10^4^ cells per mL per well in a 24-well plate. The medium was changed every 2 days. On the first and third day, the medium was removed from the flask and the pre-warmed CellTracker™ green (C7025 Invitrogen) dye working solution was added and incubated for 30 min in CO2 incubator. The dye working solution was then replaced with fresh, pre-warmed medium and the cells incubated for another 30 min at 37 °C. The cell morphology was observed under laser scanning confocal microscope (LSCM; TCS SP2; Leica, Germany). The attachment of MC3T3-E1 cells and synthesized biomaterial was observed using field-emission scanning electron microscopy (SEM, Hitachi S4800, Hitachi Ltd., Tokyo, Japan).

## Conclusion

The biomaterial which consists of GO, AG and HA was synthesized in this study with an electrophoresis system to accelerate the fabrication process. The synthesized biomaterial with 10 mA/10 h treatment of electrophoresis and addition of 1.0 wt% GO showed promising results in antibacterial property and MC3T3-E1 cells initial attachment. We concluded that the synthesized biomaterial consists of GO/AG/HA is a good candidate for BTE.
